# The impact of hospital attendance on COVID-19 infection in cancer patients: an assessment of data from Guy's Cancer

**DOI:** 10.2217/fon-2021-1329

**Published:** 2022-01-17

**Authors:** Kathryn Tremble, Louis Fox, Charlotte Moss, Beth Russell, Haleema Aljazzaf, Finola Higgins, Bill Dann, Vikash Jogia, Graham Roberts, Anne Rigg, Saoirse Dolly, Mieke Van Hemelrijck

**Affiliations:** ^1^Guy's & St Thomas' NHS Foundation Trust (GSTT), London, SE1 9RT, UK; ^2^Translational Oncology & Urology Research (TOUR), King's College London, London, SE1 9RT, UK; ^3^South East London Cancer Alliance, London, SE1 9RT, UK

**Keywords:** alpha variant, asymptomatic, cancer, COVID-19, testing

## Abstract

**Objective:** The authors monitored positivity rates of asymptomatic SARS-CoV-2 tests during the second wave of COVID-19 at Guy's Cancer Centre. **Methods:** Logistic regression was used to investigate factors associated with asymptomatic COVID positivity rates between 1 December 2020 and 28 February 2021 (n = 1346). **Results:** Living 20–40 km and 40–60 km from the alpha variant was associated with a reduced chance of a positive SARS-CoV-2 test compared with 0–20 km (odds ratio [OR]: 0.20; CI: 0.07–0.53 and OR: 0.38; CI: 0.15–0.98, respectively). An increased number of tests was associated with an increased chance of a positive SARS-CoV-2 test (OR: 1.10; CI: 1.04–1.16). **Conclusion:** The COVID-19 positivity rate of asymptomatic cancer patients is partly due to increased testing, with some contribution from the proximity of the patient population to the epicenter of the alpha variant.

## Lay abstract

The UK's second wave of COVID-19 was partly driven by the emergence of the alpha variant in the southeast of England in November 2020, spreading farther to become the predominant variant across England in December 2020. The alpha variant is associated with a greater transmissibility rate, posing an increased risk to the vulnerable population. This raised concerns about the welfare of cancer patients, as the disease and its treatment can lower one's ability to fight infection. This resulted in some cancer treatments being interrupted or stopped on the grounds of clinical safety and some follow-up care being disrupted.

In order to investigate the factors associated with asymptomatic COVID-19 positivity rates between 1 December 2020 and 28 February 2021, the authors gathered information on the number of tests taken per cancer patient at Guy's and extracted data from Guy's approved research database, which houses all routinely collected clinical data on cancer patients. This included demographic data, such as post code and age, as well as number of visits to the hospital.

From their analysis, the authors concluded that living closer to the epicenter of the alpha variant was associated with a high positivity rate; also, the more tests taken, the more likely the patients are to test positive. Therefore, the authors can conclude that attending the hospital does not increase the risk of transmission.

From September 2020, the number of COVID-19 infections started increasing in the United Kingdom (UK) as the country entered a ‘second wave' of COVID-19 infections. The UK's second wave of COVID-19 was partly driven by the alpha variant (also known as the B.1.1.7 COVID variant, the Kent variant and VOC 202012/01) [1]. The alpha variant was first detected on 20 September 2020 and is associated with greater transmissibility than the previously predominant B.1.117 variant [2]. It became the predominant variant in the southeast of England in November 2020, spreading farther to become the predominant variant across England in December 2020 [1,3].

The protection of people with cancer from COVID-19 infection has been a concern throughout the pandemic. Many cancer patients are immunosuppressed, either as a direct result of their cancer or due to their cancer treatments. Therefore, some cancer patients were identified as vulnerable to COVID-19 and its complications and were advised to shield – in particular, those undergoing chemotherapy or immunotherapy and those with hematological cancers [4]. Many cancer centers reduced their in-person delivery of care during the COVID-19 pandemic, partly to reduce the spread of COVID-19 in this vulnerable population [5,6,7]. As in-person cancer care increases back to pre-pandemic levels, it is important to understand the risks of iatrogenic spread of COVID-19 to this clinical population [8].

Guy's Cancer Centre sees around 8800 patients per year from across London and the southeast of England. Patients attending the hospital undergo frequent asymptomatic COVID-19 screening, particularly those undergoing active treatment for their cancers. To identify the factors contributing to asymptomatic positive cases in cancer patients, the authors monitored the positivity rates of cancer patients attending the Centre during the second wave.

## Methods

### Asymptomatic COVID-19 swab rates

Asymptomatic COVID-19 swab rates were monitored and compared with government data on overall COVID-19 positivity rates by region in the UK [9]. Data were averaged over a 14-day period.

### Study population

Data were extracted from the Guy's Cancer Cohort, a research ethics committee-approved research database of all routinely collected clinical data of cancer patients at Guy's and St Thomas' NHS Foundation Trust (reference number: 18/NW/0297). Data were collected on all patients with a cancer diagnosis who had a valid asymptomatic COVID-19 test at Guy's and St Thomas' NHS Foundation Trust between 1 December 2020 and 28 February 2021. The time frame studied encompassed the accelerating rates of infection during December 2020, the peak of the second wave in January 2021 and a portion of the tail after the peak in February 2021. Demographic data collected included age, post code, ethnicity and sex. Clinical data included date and result of all asymptomatic SARS-CoV-2 PCR tests performed between 1 December 2020 and 28 February 2021; date of cancer diagnosis; and number of visits to Guy's and St Thomas' NHS Foundation Trust.

### Analysis

Descriptive statistics were collated on the study population. Patients who had multiple asymptomatic COVID-19 tests during the time period were assigned as COVID-19 positive if at least one of these tests was positive. Based on the data available, the authors formulated some hypotheses to examine the likelihood that positive COVID-19 tests could be attributed to attendance at Guy's Cancer Centre. They were particularly interested in the number of attendances at the Centre, the number of tests undergone (i.e., a detection bias in the data) and the potential influence of the alpha variant of SARS-CoV-2, the epicenter of which appears to have been less than 50 km from Guy's Cancer Centre. To test these hypotheses, binary logistic regression was performed to assess the association between the following exposure variables and the SARS-CoV-2 PCR results.

- Number of attendances at the hospital between 1 December 2020 and 28 February 2021; 0–4 attendances was assigned as the reference category;
- Distance of home post code from the assumed epicenter of the alpha variant outbreak (direct distance as the crow flies); the alpha variant was assigned as post code ME4 4TR, based on the high incidence of the alpha variant in Medway local authority in late November/early December [3]; 0–20 km away from the alpha variant was assigned as the reference category;
- Number of asymptomatic COVID tests (continuous).

## Results

### Asymptomatic COVID-19 positivity rates were similar to community positivity rates

**Figure 1.**
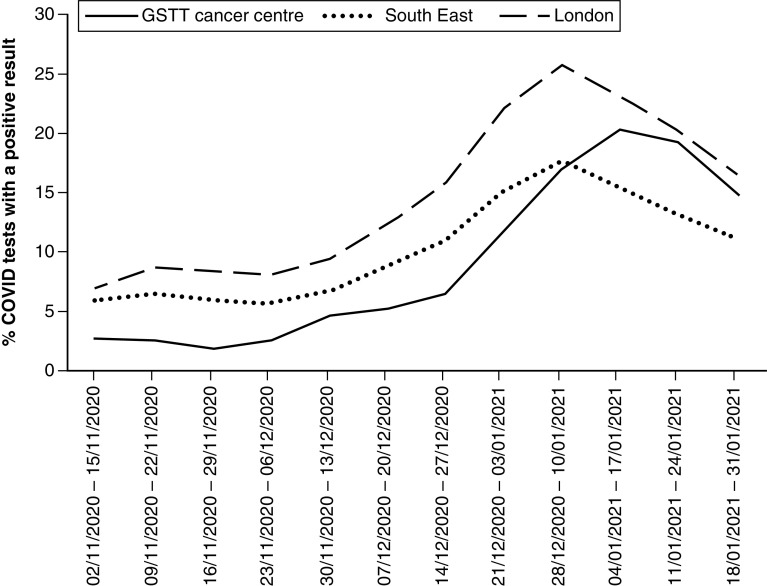
Percentage of COVID swabs with a positive result during each 2-week period at Guy's Cancer Centre and the regions of the southeast and London. GSTT: Guy's and St Thomas' NHS Foundation Trust.

The positivity rate of asymptomatic SARS-CoV-2 swab results performed in Guy's Cancer Centre peaked between 4 and 17 January 2021 at 20.3% (Figure 1). This compares to a peak of 25.8% in London and 17.7% across the southeast of England during 28 December to 10 January 2021 (Figure 1).

### Characteristics of the study population

To investigate the factors contributing to asymptomatic COVID-19 positivity rates in cancer patients, the authors identified 1346 cancer patients who had a valid asymptomatic COVID-19 test between 1 December 2020 and 28 February 2021 (Table 1); 6.5% of the patients had at least one positive COVID-19 test result. There were marginally more females than males (51.3 vs 48.7%). The average age of the patient population was 63.4 years. There was no statistically significant association between the odds of having a positive asymptomatic SARS-CoV-2 test and sex, ethnicity or age (Table 2).

**Table 1.** Characteristics of the cancer patients who received an asymptomatic COVID test between December 2020 and 28 February 2021.

	Overall population		COVID-negative		COVID-positive	
	n	%	n	%	n	%
Total	1,346	-	1,258	93.5%	88	6.5%
Gender						
Male	656	48.7%	611	48.6%	45	51.1%
Female	690	51.3%	647	51.4%	43	48.9%
Ethnicity						
White	733	54.5%	683	54.3%	50	56.8%
Asian	31	2.3%	27	2.1%	4	4.5%
Black	143	10.6%	135	10.7%	8	9.1%
Mixed	17	1.3%	15	1.2%	2	2.3%
Other	54	4.0%	51	4.1%	3	3.4%
Unknown	368	27.3%	347	27.6%	21	23.9%
Age						
<50 years	215	16.0%	200	15.9%	15	17.0%
50–59 years	300	22.3%	279	22.2%	21	23.9%
60–69 years	368	27.3%	348	27.7%	20	22.7%
70–79 years	330	24.5%	311	24.7%	19	21.6%
80 years	133	9.9%	120	9.5%	13	14.8%

**Table 2.** Odds ratios and 95% CIs for the effects of the following demographic factors on the odds of having a positive COVID-19 swab result.

	Odds ratio	95% CI
Gender		
Male	1.00	Ref
Female	0.90	0.59–1.39
Ethnicity		
White	1.00	Ref
Asian	2.02	0.68–6.01
Black	0.81	0.38–1.75
Mixed	1.82	0.41–8.19
Other	0.80	0.24–2.67
Unknown	0.83	0.49–1.40
Age	1.00	0.99–1.02

### Number of hospital attendances was not associated with an increased chance of having a positive SARS-CoV-2 result

**Figure 2.**
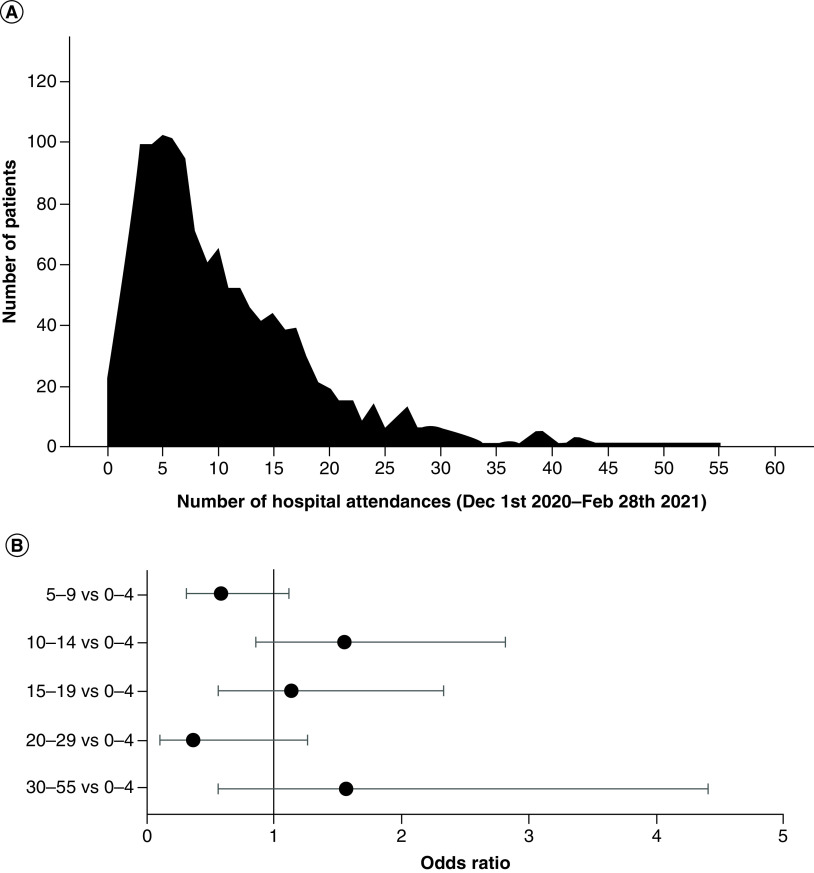
(A) Distribution of number of hospital attendances for each patient within the cohort and (B) odds ratios and 95% CIs for the effect of number of hospital attendances on the odds of having a positive asymptomatic SARS-CoV-2 PCR. Model was adjusted for sex, ethnicity and age.

The authors investigated whether attending their hospital on more occasions was associated with a higher chance of a positive asymptomatic SARS-CoV-2 test result. The number of hospital attendances for each patient between 1 December 2020 and 28 February 2021 varied from 0 to 55, with a median of eight attendances (Figure 2A). There was no statistically significant association between number of attendances and odds of having a positive asymptomatic SARS-CoV-2 test (Figure 2B).

### Living closer to the areas with high early alpha variant incidence is associated with a higher chance of having a positive SARS-CoV-2 result

**Figure 3.**
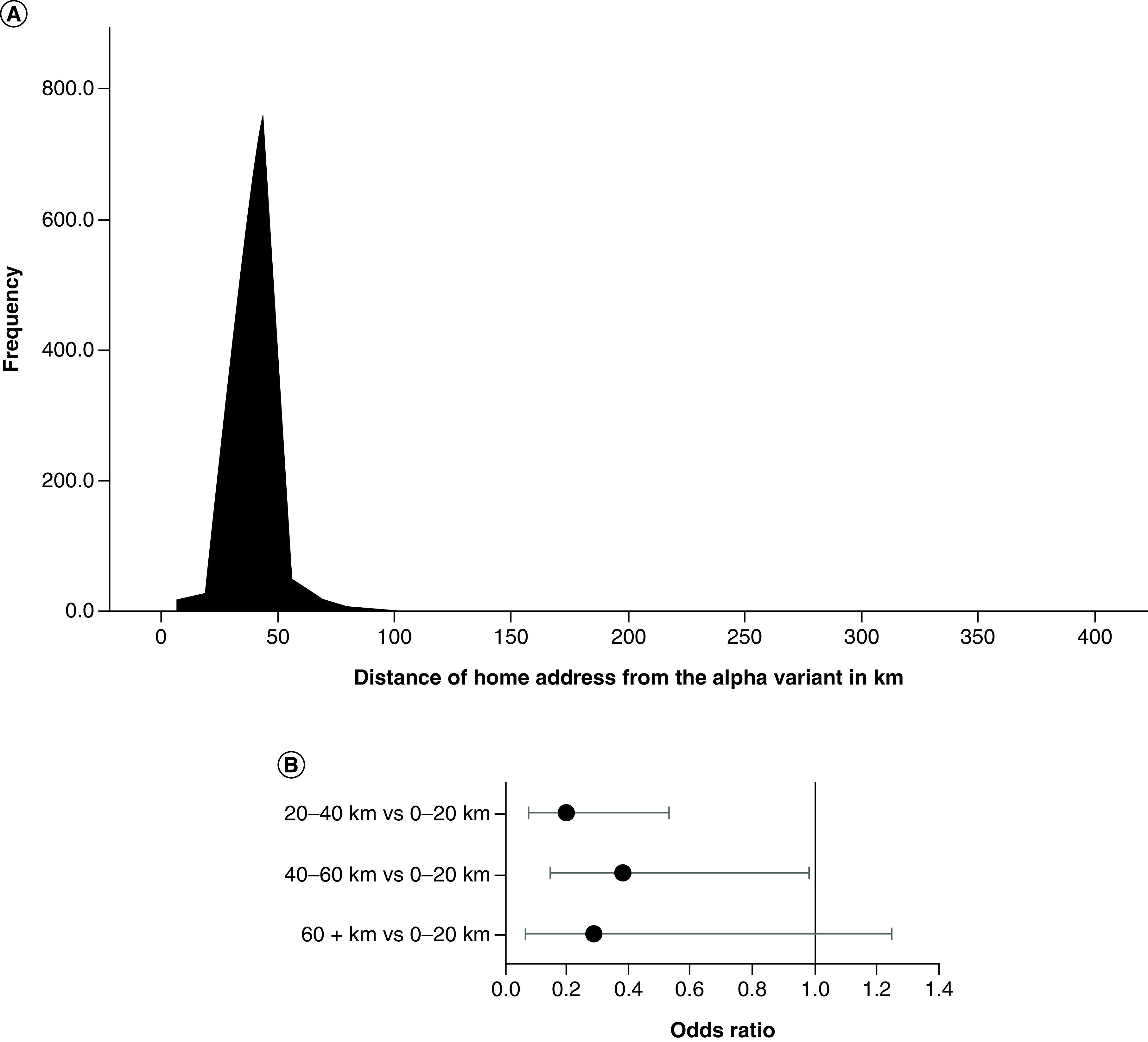
(A) Distribution of distances of patients' post codes from the area of high early incidence of the alpha variant and (B) odds ratios and 95% CIs for the effect of distance from the alpha variant in kilometers on the odds of having a positive asymptomatic SARS-CoV-2 PCR. Model was adjusted for age, sex and ethnicity.

The patient population served by Guy's Cancer Centre predominantly lives in London and the southeast of England, including areas with high early incidence of the alpha variant. The authors therefore investigated whether living closer to the alpha variant was associated with a greater COVID positivity rate. The distance of the patient's home post code from the area of high early alpha variant incidence varied from 1 km to 388 km, with a median of 40 km (Figure 3A). Living >20 km away from the areas of high incidence of the alpha variant was associated with reduced odds of having a positive asymptomatic SARS-CoV-2 test result compared with living 0–20 km away, with 20–40 km and 40–60 km reaching statistical significance (OR: 0.20; CI: 0.07–0.53 and OR: 0.38; CI: 0.15–0.98, respectively) (Figure 3B).

### Increased number of asymptomatic SARS-CoV-2 tests is associated with a higher chance of having a positive SARS-CoV-2 test result

**Figure 4.**
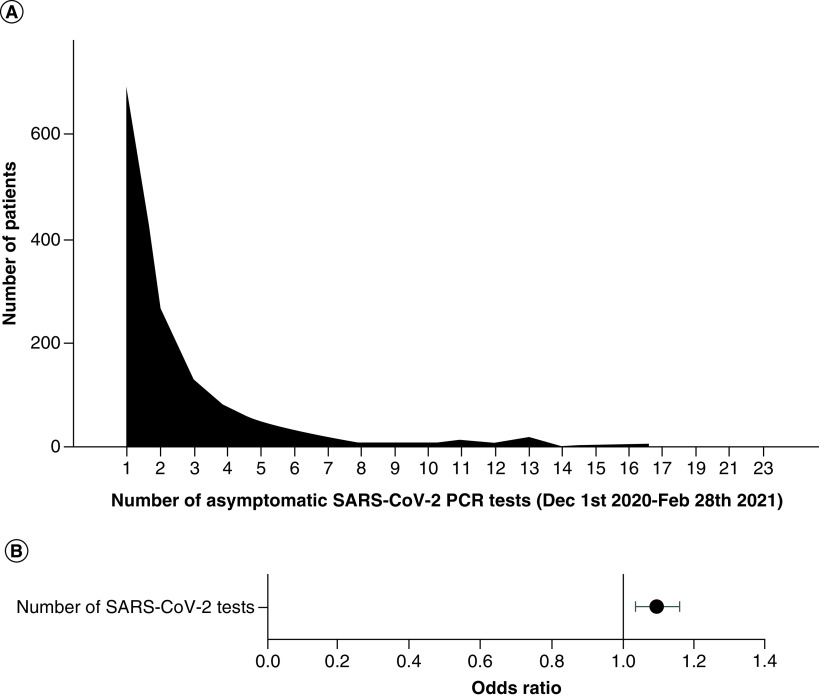
(A) Distribution of number of asymptomatic SARS-CoV-2 tests performed for the patients between 1 December 2020 and 28 February 2021 and (B) odds ratios and 95% CIs for the effect of number of asymptomatic SARS-CoV-2 PCR tests on the odds of having a positive asymptomatic SARS-CoV-2 PCR. Model was adjusted for sex, ethnicity and age.

The authors sought to investigate whether having more asymptomatic COVID-19 tests increased the odds of having a positive asymptomatic SARS-CoV-2 result. The number of asymptomatic SARS-CoV-2 tests the patients had between 1 December 2020 and 28 February 2021 varied from 1 to 23, with a median of 1 (Figure 4A). An increased number of asymptomatic SARS-CoV-2 tests was statistically significantly associated with greater odds of having a positive asymptomatic SARS-CoV-2 result, with relative odds of testing positive increasing by an estimated 10% with each additional test (OR: 1.10; CI: 1.04–1.16) (Figure 4B).

## Discussion

The authors' analysis found that attending Guy's and St Thomas' Hospital Foundation Trust more frequently is not associated with having a higher chance of COVID-19 for cancer patients. Patients living within 20 km of the areas with high incidence of the alpha variant in early December had a higher risk of COVID-19 than those living farther than 20 km away. Patients who had more frequent asymptomatic SARS-CoV-2 testing had a higher chance of testing positive for COVID-19.

There have been concerns around iatrogenic spread of COVID-19 across all specialties within healthcare, with some clinical care being delayed during the pandemic and some being transferred to virtual/telephone settings to reduce patient in-person attendances [5,6,7]. This has likely contributed to the delay in cancer diagnoses and a higher stage of cancer at diagnosis [10,11]. This analysis shows that attending the hospital more frequently does not increase the risk of having a positive COVID-19 result. This suggests that the current personal protective equipment (PPE) and social distancing guidelines are effective and supports the continued in-person treatment of cancer patients, which will be crucial in improving cancer outcomes and treating the patients who have had their treatment delayed by the COVID-19 pandemic.

The alpha variant was initially found in the Kent/London region of the UK and spread across the UK and globally, partially driving the second wave of COVID-19 in the UK [1]. Guy's Cancer Centre serves patients living in the southeast of England, including Kent and London. These data support the current understanding of the spread of the alpha variant, with a higher risk of having a positive SARS-CoV-2 swab for patients living within 20 km of the alpha variant. It also demonstrates the risk that new variants bring in driving up the numbers of COVID-19 cases, as seen with more recent variants, and supports continued monitoring for new future variants [3,12]. Hence, these observations also support the current guidelines for cancer patients, recommending that they shield when possible [13]. Nevertheless, it needs to be highlighted that there is still uncertainty around the balance of threats and opportunities related to telemedicine for cancer patients [14], one of the mechanisms that support shielding as part of their clinical care pathway. Based on patients' clinical and demographic characteristics, telemedicine may not always be an option, and hence the safety of in-person hospital visits still needs to be considered.

The authors found that increased COVID-19 testing is associated with an increased chance of having a positive result. Estimates for the proportion of COVID-19 cases that are asymptomatic vary, with most studies agreeing on between 10 and 30% [15,16]. Repeated asymptomatic COVID-19 testing will pick up more of these asymptomatic cases, leading to a higher chance of having at least one positive result. Additionally, the false-positive rate will be higher in asymptomatic testing compared with symptomatic testing [17]. Therefore, the increased testing at least partially explains the high number of COVID-positive results in the authors' asymptomatic cancer population, compared with the local symptomatic community population.

This analysis is based on data from Guy's Cancer Centre, which delivers care to a large number of patients per year and serves an ethnically and economically diverse population. Therefore, it is informative for other cancer and non-cancer services to get an understanding of the risk of iatrogenic spread of COVID-19. It covers the area where the alpha variant was first discovered and, so, provides insights into the spread of the alpha variant during the second wave. This analysis may not be reflective of other healthcare settings with different PPE and social distancing guidelines. Furthermore, future studies with longer follow-up and assessment of the spread of other variants will be required to help inform clinical practice.

## Conclusion

These real-world data suggest that the relative parity in COVID-19 positivity rates observed between the authors' cancer center and the wider local community during the UK's second wave may be partially explained by increased asymptomatic testing and a skew in their patient population toward individuals residing closer to the epicenter of a SARS-CoV-2 variant of concern. Hence, these findings are supportive of the continued in-person delivery of cancer care and indicate that the PPE and social distancing guidelines currently in use at Guy's Cancer Centre are adequately managing the risks posed to cancer patients by COVID-19.

**Summary points**

- To identify factors contributing to asymptomatic positive cases of COVID-19 in cancer patients, the authors monitored the positivity rates of cancer patients attending their center during the second wave.
- This analysis found that attending Guy's and St Thomas' Hospital Foundation Trust more frequently is not associated with having a higher chance of COVID-19 for cancer patients.
- Patients living within 20 km of the areas with high incidence of the alpha variant in early December had a higher risk of COVID-19 than those living farther than 20 km away.
- Patients who had more frequent asymptomatic SARS-CoV-2 testing had a higher chance of testing positive for COVID-19.
- These findings support the continued in-person delivery of cancer care and indicate that the personal protective equipment and social distancing guidelines currently in use at Guy's Cancer Centre are adequately managing the risks posed to cancer patients by COVID-19.

## References

[1] Public Health England. Investigation of Novel SARS-CoV-2 Variant. Variant of Concern 202012/01. Technical Briefing 5. (2021).

[2] Davies NG, Abbott S, Barnard RC Estimated transmissibility and impact of SARS-CoV-2 lineage B.1.1.7 in England. Science 372(6538), eabg3055 (2021). 33658326 10.1126/science.abg3055 PMC8128288

[3] COVID-19 Genomics UK Consortium. Wellcome Sanger Institute. https://covid19.sanger.ac.uk/

[4] Public Health England. Guidance on Protecting People Most Likely to Get Very Poorly from Coronavirus (COVID-19). (.2021).

[5] Spencer K, Jones CM, Girdler R The impact of the COVID-19 pandemic on radiotherapy services in England, UK: a population-based study. Lancet Oncol. 22(3), 309–320 (2021). 33493433 10.1016/S1470-2045(20)30743-9 PMC7825861

[6] Monroy-Iglesias MJ, Tagliabue M, Dickinson H Continuity of cancer care: the surgical experience of two large cancer hubs in London and Milan. Cancers 13(7), 1597 (2021). 33808375 10.3390/cancers13071597 PMC8036608

[7] Russell B, Harris V, Dickinson H Abstract P18: radical cancer treatment is safe during COVID-19: the experience of a large London-based comprehensive cancer centre. Clin. Cancer Res. 27(Suppl. A), P18 (2021). 10.1038/s41416-022-01909-0 PMC9284490 35840733

[8] Impact of COVID-19 on urgent suspected cancer referrals and diagnostic testing. Cancer Research UK. www.cancerresearchuk.org/health-professional/diagnosis/hp-covid-19-and-cancer-hub#HP_COVID-190 (Accessed 8 September 2021)

[9] Coronavirus (COVID-19) in the UK. https://coronavirus.data.gov.uk/

[10] Morris EJA, Goldacre R, Spata E Impact of the COVID-19 pandemic on the detection and management of colorectal cancer in England: a population-based study. Lancet Gastroenterol. Hepatol. 6(3), 199–208 (2021). 33453763 10.1016/S2468-1253(21)00005-4 PMC7808901

[11] Purushotham A, Roberts G, Haire K The impact of national non-pharmaceutical interventions (‘lockdowns’) on the presentation of cancer patients. Ecancermedicalscience 15, 1180 (2021). 33777173 10.3332/ecancer.2021.1180 PMC7987492

[12] Public Health England. Investigation of Novel SARS-CoV-2 Variants of Concern (England) – Technical Briefing 11, 13 May 2021 (2021).

[13] Cancer Research UK. Coronavirus shielding advice. www.cancerresearchuk.org/about-cancer/cancer-in-general/coronavirus/shielding-advice

[14] Elkaddoum R, Haddad FG, Eid R, Kourie HR. Telemedicine for cancer patients during COVID-19 pandemic: between threats and opportunities. Future Oncol. 16(18), 1225–1227 (2020). 32356460 10.2217/fon-2020-0324 PMC7202358

[15] He J, Guo Y, Mao R, Zhang J. Proportion of asymptomatic coronavirus disease 2019: a systematic review and meta-analysis. J. Med. Virol. 93(2), 820–830 (2021). 32691881 10.1002/jmv.26326 PMC7404334

[16] Beale S, Hayward A, Shallcross L, Aldridge RW, Fragaszy E. A rapid review and meta-analysis of the asymptomatic proportion of PCR-confirmed SARS-CoV-2 infections in community settings. Wellcome Open Research 5, 266 (2020).

[17] Hopkins S. COVID-19: Reintroducing confirmatory PCR testing. Public Health England. https://publichealthmatters.blog.gov.uk/2021/03/30/covid-19-reintroducing-confirmatory-pcr-testing/

